# Serotype Competence and Penicillin Resistance in *Streptococcus pneumoniae*

**DOI:** 10.3201/eid1211.060414

**Published:** 2006-11

**Authors:** Yu-Chia Hsieh, Jin-Town Wang, Wen-Sen Lee, Po-Ren Hsueh, Pei-Lan Shao, Luan-Yin Chang, Chun-Yi Lu, Chin-Yun Lee, Fu-Yuan Huang, Li-Min Huang

**Affiliations:** * Taipei Medical University-WanFang Hospital, Taipei, Taiwan;; † National Taiwan University Hospital, Taipei, Taiwan;; ‡ Mackay Memorial Hospital, Taipei, Taiwan

**Keywords:** *Streptococcus pneumoniae*, competence, serotype, antimicrobial resistance, research

## Abstract

From 2003 to 2005, we prospectively collected 118 isolates of pneumococci belonging to 7 serotypes to investigate their competence under the influence of the synthetic competence-stimulating peptides. The degree of competence of the various serotypes differed significantly. Serotype 6B had the highest competence, followed by serotypes 14, 19F, 9V, 23F, 3, and 18C. Isolates belonging to serotype 6B had greater genetic diversity than isolates belonging to serotype 3, which has high genetic clustering. Isolates belonging to serotypes 3 and 18C that were 100% sensitive to penicillin were significantly less competent than isolates belonging to serotypes 6B, 14, 19F, 9V, and 23F, which were frequently resistant to penicillin. Under the 7-valent pneumococcal conjugate vaccine program, enhanced molecular surveillance of virulent clones with higher competence to detect serotype switching will become more important.

## Abstract 2

Enhanced molecular surveillance of virulent clones with higher competence can detect serotype switching.

Streptococcus pneumoniae, a leading cause of bacteremia, sinusitis, otitis media, bacterial meningitis, and bacterial pneumonia, causes substantial illness and death in persons worldwide (*1*). In recent decades, the increase of S. pneumoniae strains resistant to β-lactam antimicrobial drugs and other classes of antimicrobial drugs has further complicated the treatment of pneumococcal infection (*2*). Although the current introduction of conjugate pneumococcal vaccine has successfully reduced invasive pneumococcal disease caused by the vaccine serotypes and effectively decreased the spread of antimicrobial drug–resistant isolates, pneumococcal infection remains a major issue, in light of the selective pressure that has been invoked by vaccination programs (*3**,**4*). At least 2 consequences have been noted since the large-scale use of 7-valent conjugate vaccine. First, serotypes not covered by the conjugate vaccine have increased both in nasopharyngeal colonization and clinical illness (serotype replacement) (*5**,**6*). Second, serotype switching can occur through recombination in naturally transformable clones and result in the acquisition of a nonvaccine capsule (*5**,**7*). Therefore, investigating how competent pneumococci can be for genetic transformation is useful because this factor plays a role in the evolution of S. pneumoniae, especially with respect to virulence and antimicrobial drug resistance (*8**,**9*). The ability of S. pneumoniae to undergo horizontal gene transfer leads to genetic diversity and helps the organism adapt to environmental changes. Since the discovery of competence-stimulating peptide (CSP) by Havarstein et al., the detailed mechanism of competence in S. pneumoniae has gradually been clarified (*10*). However, few studies have examined the different competence levels across a range of clinical isolates (*11*). Our aims in this study were to compare competence among clinical isolates of various serotypes and to increase our understanding of the role of competence in penicillin-resistant S. pneumoniae.

## Materials and Methods

### Bacterial Isolates

From January 2003 to December 2005, a total of 118 S. pneumomiae isolates were prospectively collected for this study. These isolates were recovered from various clinical specimens obtained from patients in 6 medical centers in Taiwan. The samples were collected from areas in which pneumococcal conjugate vaccine has not been widely implemented. The hospitals included National Taiwan University Hospital, Taipei (62 isolates); Taichung Veterans General Hospital, Taichung (19 isolates); People's Republic of China Medical College Hospital, Taichung (5 isolates); National Cheng-Kung University Hospital, Tainan (3 isolates); Chang-Gung Memorial Hospital, Kaohsiung (12 isolates); and Kaohsiung Veterans General Hospital, Kaohsiung (17 isolates). Among these isolates, 24 isolates (20.2%) were recovered from normally sterile body sites (21 isolates from blood, 2 isolates from pleural fluid, and 1isolate from peritoneal fluid); the rest were isolated from respiratory tract secretions.

### Antimicrobial Drug Susceptibility Testing

The MICs of penicillin for all 118 S. pneumoniae isolates were determined by the agar dilution method and were interpreted according to the guidelines established by the Clinical and Laboratory Standards Institute (CLSI; formerly the National Committee for Clinical Laboratory Standards) (*12**,**13*). Strains with an MIC <0.06 μg/mL were defined as susceptible, i.e., penicillin-susceptible S. pneumoniae (PSSP). Strains with an MIC of 0.12–1 μg/mL were defined as intermediately resistant, i.e., penicillin-intermediate S. pneumoniae (PISP), whereas those with an MIC^3^ 2 μg/mL were penicillin-resistant S. pneumoniae (PRSP).

### Serotyping

The serotypes of isolates were determined by using the capsular swelling method (Quellung reaction). All antisera were obtained from the Statens Seruminstitut (Copenhagen, Denmark).

### PFGE Analysis

Serotype 6B, which had the highest competence, and serotypes 3 and 18C, which had the lowest competence, were selected for pulsed-field gel electrophoresis (PFGE) analysis. PFGE was performed according to the method previously described (*14*). The DNA was digested with SmaI. Bands were stained with ethidium bromide and visualized with UV light. PFGE patterns that differed by <3 bands were defined as 1 PFGE type; isolates with the same PFGE patterns indicated indistinguishable strains, and those with 2 or 3 different bands indicated closely related strains (*15*).

### Transformation of S. pneumoniae

All pneumococcal isolates were grown at 35°C in Todd–Hewitt broth supplemented with 0.5% yeast extract (THY) in static culture in the presence of 5% CO_2_. Bacteria were stored in THY and 10% glycerol. Transformations were performed as described previously with modification (*8**,**16*). Briefly, early log phase S. pneumoniae cultures in THY, pH 6.8 (pH adjusted with HCl), supplemented with 1 mmol/L CaCl_2_ and 0.2% bovine serum albumin (BSA) were diluted 1:10 with THY, pH 8.0 (adjusted with NaOH), supplemented with 1 mmol/L CaCl_2_ and 0.2% BSA. Synthetic 100 ng/mL CSP-1 or CSP-2, in 10 mmol/L glucose and 10% horse serum (Sigma, Saint Louis, MO, USA) were added followed by incubation for 15 min at 37°C. Plasmid pDL278, an Escherichia coli/S. pneumoniae shuttle vector that contained the pVA380–1 basic replicon and the pUC origin of replication ( 6,733 bp) was then added (1 μg/mL), and samples were incubated for 1 h at 35°C under 5% CO_2_ before being spread on blood agar plates containing 500 μg/mL spectinomycin (*17*). Control experiments were carried out by using the same protocol without adding pDL278. The transformation frequencies were expressed as the log_10_ value of the percentage of transformed cells. Isolates with a log_10_ value of transformation frequencies less than –10 were defined as noncompetent isolates, and those larger than –4 were defined as high competent isolates. (Because the report of transformation frequency in S. pneumoniae is limited, we chose –4 as the cutoff point of high frequency based on the experience of transformation frequency in Helicobacter pylori [18].)

### Statistical Analysis

After log transformation, the data for competence (transformation frequency) exhibited a normal distribution (p>0.05). Thus, differences of competence between multiple serotypes and groups were tested by using 1-way analysis of variance with the Bonferroni method for post-hoc multiple comparisons. The Student t test was used when competence were compared between 2 groups. Linear regression was used to detect the trend of competence among the PSSP, PISP, and PRSP groups. The relationship of penicillin resistance with competence was analyzed in a logistic regression model which controlled for serotypes. χ^2^ test or Fisher exact test was used for categorical variables to test significance between groups. Correlations between competence induced by CSP-1 and CSP-2 in each serotype were determined by Pearson's correlation coefficient. A p value <0.05 was considered significant. All probabilities were 2-tailed. Data were reported as mean ± standard error of the mean (SEM) unless otherwise indicated.

## Results

We found 7 serotypes among the 118 isolates: 6B (23 strains), 14 (20 strains), 23F (26 strains), 9V (14 strains), 19F (21 strains), 3 (11 strains), and 18C (3 strains). Of the isolates, 26.3% were susceptible to penicillin; 54.2% were intermediately resistant and 19.5% were highly resistant.

### Competence, Serotype, and Genetic Heterogeneity

**Figure 1:**
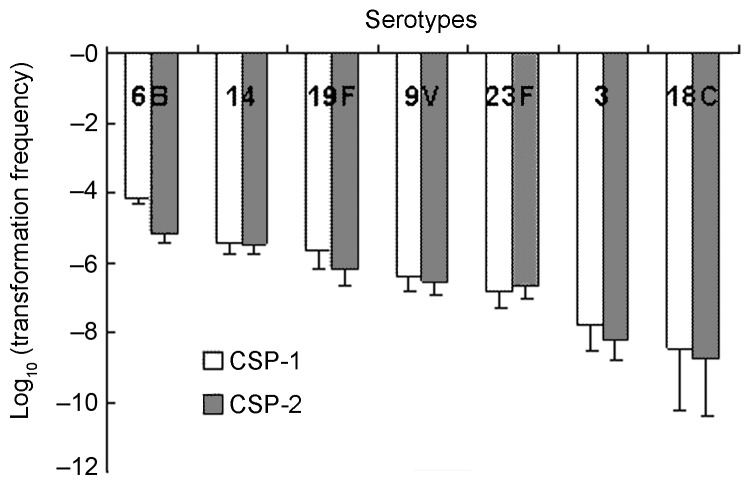
Competence (transformation frequency) induced by competence-stimulating peptide 1 (CSP-1) and CSP-2 in clinical isolates.

**Figure 2:**
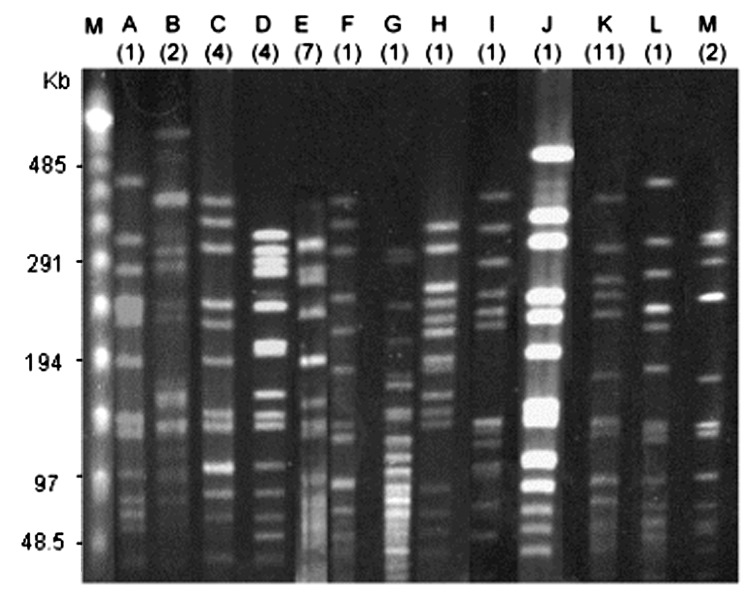
Pulsed-field gel electrophoresis (PFGE) types of 47 isolates of serotype 6B, serotype 3, and serotype 18C. Numbers in parentheses indicate the total number of isolates belonging to each PFGE type. Lane M, λ ladder; lanes A–J, 10 PFGE types found among 23 isolates of serotype 6B; lane K, 1 PFGE type found among 11 isolates of serotype 3; lanes L–M, 2 PFGE types found among 3 isolates of serotype 18C.

Figure 1 shows the log-transformed means (±SEM) of the transformation frequencies of serotypes 6B, 14, 19F, 9V, 23F, 3, and 18C induced by CSP-1 and CSP-2. In general, competence induced by CSP-1 was higher than that induced by CSP-2, except for serotype 23F. Competence induced by CSP-1 was positively correlated with competence induced by CSP-2 (Pearson's correlation coefficient 0.84, p<0.001). Significant differences of competence were found among serotypes under induction by either CSP-1 or CSP-2 (p<0.001). Serotype 6B had the highest competence, followed by 14, 19F, 9V, 23F, 3, and 18C. Under the induction of CSP-1, competence in serotype 6B (–4.1 ± 0.2) was significantly higher than for serotypes 9V (–6.4 ± 0.4), 23F (–6.8 ± 0.5), 3 (–7.8 ± 0.7) and 18C (–8.4 ± 1.8); similarly, competence in serotype 14 (–5.4 ± 0.3) was significantly higher than for serotype 3 (p<0.05 by post-hoc analysis). Under the induction of CSP-2, competence in serotype 6B (–5.2 ± 0.3) was significantly higher than those in serotypes 23F (–6.7 ± 0.4), 3 (–8.1 ± 0.6), and 18C (–8.8 ± 1.6). Competence in serotypes 14 (–5.5 ± 0.3) and 19F (–6.2 ± 0.5) was significantly higher than for serotype 3, and competence in serotype 14 was significantly higher than for serotype 18C (p<0.05 by post-hoc analysis). The 23 isolates expressing serotype 6B displayed a high level of genetic diversity; this was illustrated by their division into 10 PFGE patterns (Figure 2). Among them, 1 main PFGE type accounted for 30.4% (7/23) of all isolates of serotype 6B. These 7 strains were closely related. In contrast, the 11 isolates expressing serotype 3 were genetically indistinguishable, which showed only 1 PFGE pattern. The result indicated that this serotype was highly clonal in Taiwan (Figure 2). The 3 isolates expressing serotype 18C had 2 PFGE types. Twenty-two isolates had a competence higher than 10^–4^ under the induction of CSP-1: 11 isolates of 6B, 4 isolates of 14, 4 isolates of 19F, and 3 isolates of 23F. Under the induction of CSP-1, isolates belonging to serotypes 6B, 14, 19F, and 23F were significantly associated with high competence when compared with isolates belonging to serotypes 9V, 3, and 18C (24.4%, 22/90 vs 0%, 0/28; p = 0.002). Six isolates had competence higher than 10^–4^ under the induction of CSP-2: 2 isolates of 6B, 3 isolates of 14, and 1 isolate of 23F. Under the induction of CSP-2, isolates belonging to serotypes 6B, 14, 19F, and 23F were not associated with high competence compared with isolates belonging to serotypes 9V, 3, and 18C (6.7%, 6/90 vs. 0%, 0/28; p = 0.3). Among all the 118 isolates, 111 (94.1%) became competent after the induction with CSP-1, and 112 (94.9%) became competent after the induction with CSP-2. Two isolates of 19F, 1 isolate of 23F, 1 isolate of serotype 18C, and 2 isolate of serotype 3 were noncompetent with either CSP-1 or CSP-2.

### Competence and Penicillin Resistance

**Figure 3:**
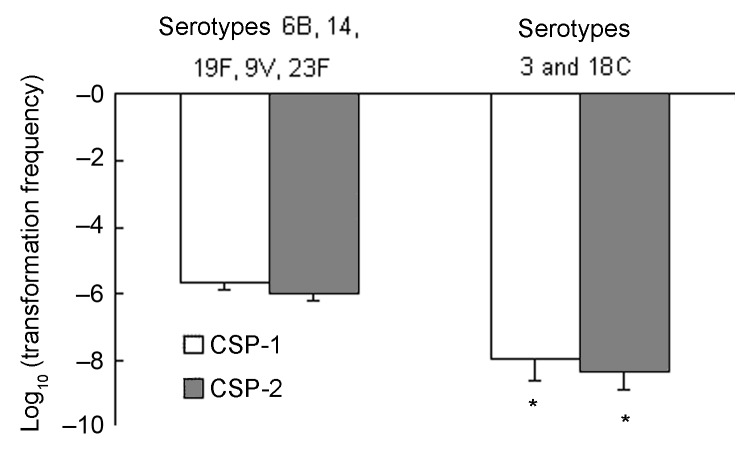
Competence (transformation frequency) induced by competence-stimulating peptide 1 (CSP-1) and CSP-2 between isolates belonging to serotypes 6B, 14, 19F, 9V, and 23F, and isolates belonging to serotypes 3 and 18C (*, p<0.05).

**Figure 4:**
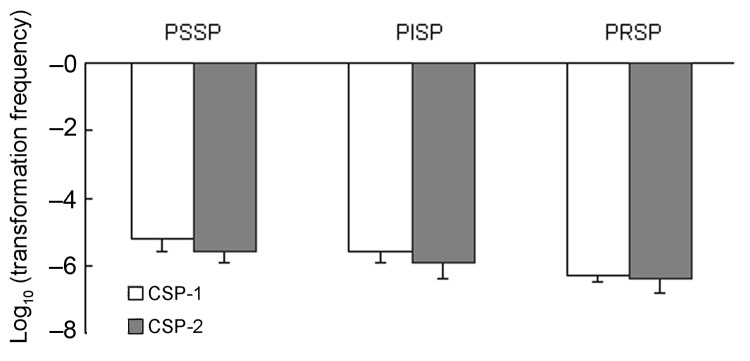
Competence (transformation frequency) induced by competence-stimulating peptide 1 (CSP-1) and CSP-2 among 3 groups: PSSP, PISP, and PRSP. PSSP, penicillin-susceptible *Streptococcus pneumoniae*; PISP, penicillin-intermediate *S. pneumoniae*; PRSP, penicillin-resistant *S. pneumoniae*.

The distributions of PSSP, PISP, and PRSP in each serotype were significantly different (p = 0.001) (Table). The proportions of penicillin-susceptibility in serotypes 6B, 14, 19F, 9V and 23F were 8.7%, 35%, 19%, 21.4%, and 3.8%, respectively. (Table). While in serotypes 3 and 18C, the proportions of penicillin-susceptibility were 100%. Under the induction of either CSP-1 or CSP-2, isolates belonging to serotypes 3 and 18C that were not resistant against penicillin were significantly less competent than isolates belonging to serotypes 6B, 14, 19F, 9V, and 23F, which were frequently resistant to penicillin (p<0.001 for CSP-1, p<0.001 for CSP-2 by the Student t test) (Figure 3). Among all isolates belonging to serotype 6B, 14, 19F, 9V, and 23F, the proportions of PSSP, PISP, and PRSP were 16.3%, 61.5%, and 22.2%. Competence between PSSP, PISP, and PRSP was not significantly different (p = 0.2 for CSP-1, p = 0.3 for CSP-2) (Figure 4). By using the linear regression test to test for trend, competence was not significantly correlated with increasing penicillin resistance among isolates belonging to serotypes 6B, 14, 19F, 9V, and 23F (p = 0.7 for CSP-1, p = 0.3 for CSP-2). In a logistic regression model that controlled for serotypes, competence was not significantly correlated with penicillin resistance (odds ratio 0.8, p = 0.3, 95% confidence interval [CI] 0.58–1.17 for CSP-1; odds ratio 0.9, p = 0.4, 95% CI 0.58–1.25 for CSP-2).

**Table:** Percentage of PSSP, PISP, and PRSP in each serotype*

Degree of susceptibility	Serotype						
	6B, n = 23 (%)	14, n = 20 (%)	19F, n = 21 (%)	9V, n = 14 (%)	23F, n = 26 (%)	3, n = 11 (%)	18C, n = 3 (%)
PSSP (MIC £0.06 μg/mL)	2 (8.7)	7 (35)	4 (19)	3 (21.4)	1 (3.8)	11 (100)	3 (100)
PISP (MIC 0.1–1 μg/mL)	18 (78.3)	10 (50)	12 (57.1)	5 (35.7)	19 (73.1)	0	0
PRSP (MIC >2 μg/mL)	3 (13)	3 (15)	5 (23.8)	6 (42.9)	6 (23.1)	0	0

*PSSP, penicillin-susceptible *Streptococcus pneumoniae*; PISP, penicillin-intermediate *S. pneumoniae*; PRSP, penicillin-resistant *S. pneumoniae*.

## Discussion

S. pneumoniae was the first pathogen to demonstrate the phenomenon of transformation (*19*). In 1944, Avery et al. proved that the genetic material in bacterial cells was DNA by using a transformation model in S. pneumoniae (*20*). Natural competence for genetic transformation in S. pneumoniae is mediated by a quorum sensing-regulated system. CSP, a heptadecapeptide pheromone, induces competence in growing cells at a critical cell density by activating the 2-component signal transduction system comDE (*10*). However, spontaneous competence has been observed only in some rough laboratory strains. Most clinical encapsulated isolates do not show competence unless synthetic CSP is added (*11*). In this study, we examined the levels of competence of various clinical isolates with the aid of synthetic CSP to gain insight into the association between competence and serotype and penicillin resistance.

Our results showed that different serotypes of S. pneumoniae possess different levels of competence. Serotype 6B was the most competent, consistent with our findings that these strains had high genetic diversity. On the other hand, serotypes 3 were less competent, consistent with our findings that these strains had low genetic heterogeneity. Large amounts of capsular polysaccharide have been reported to have an inhibitory effect on transformation in S. pneumoniae (*21*), and therefore relatively rich amounts of capsular polysaccharide in serotype 3 and 18C may block uptake of foreign DNA. Finding extremely low competence in serotype 3 could explain the limited genetic heterogeneity in serotype 3, which has also been observed in Canada, the United Kingdom, and the Netherlands (*22*). Serotype 3 was an infrequent pathogen among childhood pneumococcal diseases before the conjugate pneumococcal vaccination was implemented, even though the serotype is highly virulent (*22**,**23*); however, it is emerging as an important pathogen after the implementation of conjugate pneumococcal vaccination programs (*24**,**25*). We thought that pneumococcal conjugate vaccine could expand to include serotype 3 because of its high virulence and limited capacity to facilitate capsular transformation through horizontal DNA transfer of serotype 3 to better prevent severe childhood pneumococcal disease.

S. pneumoniae acquires mosaic penicillin-binding protein (PBP) genes from other Streptococcus species through a transformation to become penicillin-resistant (*26*). Isolates belonging to serotypes 3 and 18C are too low in competence to have a chance of acquiring the penicillin-resistance gene. Therefore, serotypes 3 and 18C are rarely resistant to penicillin (*27*). Among isolates belonging to 6B, 14, 19F, 9V, and 23F, no significant difference and association between competence and different level of penicillin resistance were found. Our explanations based on this finding are as follows: 1) A high level of competence is needed for acquiring the penicillin-resistance gene. But, being more competent does not necessarily result in being penicillin resistant or in having a higher MIC of penicillin in isolates of serotypes 6B, 14, 19F, 9V, and 23F. 2) The development of penicillin resistance in S. pneumoniae is a multistep process. In addition to PBP, mutation or other non-PBP elements are also important, especially in the formation of PRSP (*28**,**29*). 3) After acquiring the penicillin-resistance gene, originally competent isolates might lose genetic components important for competence during high frequency of genetic transformation, resulting in isolates that are not competent.

Serotypes with higher competence are more likely to undergo recombinational exchanges to produce a new serotype or penicillin-resistant variant (*30*). The limitation of our study is examining competence for genetic transformation in S. pneumoniae by using in vitro assay. We are not sure if this method can reflect the real transformation capacity of S. pneumoniae in vivo. In our study, isolates belonging to serotypes 6B, 14, 19F, and 23F were associated with high competence, >10^–4^, a finding that was in line with earlier studies that observed that serotype 6B, 14, 19F, and 23F frequently showed in vivo capsular transformation and related to be penicillin-resistant clinical isolates (*7**,**31**–**34*). The only exception is the relatively low competence of our 14 isolates belonging to serotype 9V, in which international Spain^9V^ had usually been reported to have in vivo capsular transformation (*35**,**36*). Certain serotypes that are frequently involved in capsular switching and penicillin resistance attributable to high competence should be further studied. To our knowledge, this is the first investigation into the relationship of competence and clinical characteristics in S. pneumoniae. After the introduction of the 7-valent pneumococcal conjugate vaccine, particular strains with genetic advantage may change their capsules from vaccine serotypes to nonvaccine serotype through capsular transformation (*5**,**7*). We suggest that enhanced surveillance of virulent clone with higher competence should allow the detection of serotype switching. This would be valuable for the long-term effectiveness of the conjugate vaccine.
